# Advantages and challenges of using arterial spin labelling MRI to monitor cerebral blood flow in multi-centre clinical trials of neurodegenerative disease: Experience from the RADAR study

**DOI:** 10.1016/j.cccb.2024.100376

**Published:** 2025-01-01

**Authors:** Lina Jarutyte, Jan Petr, Nicholas Turner, Patrick G. Kehoe, Henk-Jan Mutsaerts, David L. Thomas

**Affiliations:** a School of Psychological Science, University of Bristol, Bristol, UK; b Nuffield Department of Population Health, University of Oxford, Oxford, UK; c Helmholtz-Zentrum Dresden-Rossendorf, Institute of Radiopharmaceutical Cancer Research, Dresden, Germany; d Bristol Medical School, University of Bristol, Bristol, UK; e Elizabeth Blackwell Institute for Health Research, University of Bristol; f Department of Radiology and Nuclear Medicine, Amsterdam Neuroscience, Amsterdam University Medical Center, Amsterdam, the Netherlands; g Department of Brain Repair and Rehabilitation, UCL Queen Square Institute of Neurology, University College London, London, UK

**Keywords:** Cerebral blood flow, Arterial spin labelling, MRI, Multi-centre randomised controlled trials, Alzheimer's disease, Hypertension, Blood pressure, Angiotensin

## Abstract

Arterial spin labelling (ASL) enables non-invasive quantification of regional brain perfusion using MRI. ASL was used in the Reducing Pathology in Alzheimer's Disease through Angiotensin TaRgeting (RADAR) multi-centre trial to pilot the assessment of the effects of the anti-hypertension drug losartan on cerebral blood flow (CBF). In the multi-centre setting, disparities in ASL implementation on scanners from different manufacturers lead to inherent differences in measured CBF and its associated parameters (e.g. spatial coefficient of variation (sCoV) of CBF, a proxy of arterial arrival times). In addition, differences in ASL acquisition parameter settings can also affect the measured quantitative perfusion values. In this study, we used data from the RADAR cohort as a case study to evaluate the site-dependent systematic differences of CBF and sCoV, and show that variations in the readout module (2D or 3D) and the post-labelling delay acquisition parameter introduced artifactual group differences. When accounting for this effect in data analysis, we show that it is still possible to combine ASL data across sites to observe the expected relationships between grey matter CBF and cognitive scores. In summary, ASL can provide useful information relating to CBF difference in multi-centre therapeutic trials, but care must be taken in data analysis to account for the inevitable inter-site differences in scanner type and acquisition protocol.

## Highlights

• Arterial spin labelling (ASL) MRI measures cerebral blood flow (CBF) non-invasively.
• CBF has been suggested as an outcome measure in neurological clinical trials.
• In multi-centre studies, measured ASL CBF depends on scanner hardware and software.
• Site-dependent differences can successfully be accounted for in data analysis.
• ASL is well suited as an outcome measure in large multi-centre therapeutic trials.

## Introduction

Alzheimer's Disease (AD) is the most common form of dementia, with approximately 55 million people living with the disease worldwide [1]. AD is traditionally characterised by the build-up of amyloid and tau proteins in the brain, with recent breakthroughs using anti-amyloid monoclonal antibody therapies providing the first convincing possibility of disease-modifying treatments [2]. In addition, vascular risk factors have long been recognized and shown to play an important exacerbating role in AD pathogenesis, and both midlife ([3,4]) and late life [5] hypertension are known to increase the risk of dementia developing later in life.

Motivated by these links between vascular risk factors and likely cerebrovascular dysfunction in AD, the Reducing Pathology in Alzheimer's Disease through Angiotensin TaRgeting (RADAR) trial [6] was designed to assess efficacy of the angiotensin type 1 receptor antagonist (AT1RA) losartan to reduce brain atrophy and cognitive decline in AD. Losartan was the first in a new class of blood pressure lowering drugs that inhibit the signalling of angiotensin II. Losartan was designed as a treatment for hypertension but has also previously been observed to be associated with a reduced incidence of AD ([7,8]). Losartan has been shown to improve cerebral blood flow (CBF) [9], and in low doses (i.e., not sufficient to affect blood pressure (BP)), reduces pathology and improves cognitive performance in transgenic mouse models of AD [10]. The RADAR study was a multi-centre, phase II, two-arm, double-blind, placebo-controlled randomised trial of high dose (100mg) daily losartan in patients with mild-to-moderate AD. The primary outcome of the study was the rate of whole brain atrophy, measured using T1-weighted structural MRI. In addition, arterial spin labelling (ASL) was included in the MRI protocol as a nested exploratory study, to provide a direct measure of changes in brain perfusion associated with losartan administration in this cohort. In particular, this was intended to provide the opportunity to identify any potentially unfavourable reductions in CBF associated with the lowering of BP, and test its scope for analysis with other outcome measures in the study.

ASL is a non-invasive MRI method for mapping CBF in the brain [11]. It provides voxel-wise CBF estimates in quantitative physiological units of mL/100 g/min, and requires no contrast agent injection or other intervention. Although it is not provided as a standard sequence by some vendors, and therefore may incur extra cost by needing to be purchased as an ‘optional extra’, it is otherwise ideally suited for use in clinical research studies and trials of neurodegenerative disease, as any effects of treatments on CBF can be monitored longitudinally with arbitrary regularity ([12,13]). For this reason, and given the vasomodulatory properties of the intervention (losartan) being investigated, it was chosen for inclusion in the RADAR MRI protocol. The multi-centre nature of the RADAR trial necessitated a range of MRI scanner types to be used, with the three main scanner manufacturers (GE, Philips and Siemens) all represented. While for most MRI sequence types (e.g., structural 3D T1-weighted or 3D FLAIR imaging) this variation in scanner type has a limited impact on image contrast and compatibility, for ASL it has more profound implications ([14,15]). This is due to the existence of several different subtypes of ASL, including pulsed ASL (PASL) and pseudo-continuous ASL (PCASL), each of which can be implemented with different rapid readout acquisition schemes (e.g., 2D echo-planar imaging (EPI) [16] or 3D gradient and spin echo (GRASE) ([17,18]) imaging). Each ASL subtype and readout scheme generates images with slightly different characteristics and contrast [19]. Unfortunately, each manufacturer provides a different ASL subtype/readout combination, resulting in large discrepancies in the ASL data acquired in group studies that combine data from different scanners [15]. Alongside this, mismatches of the acquisition parameters, even for the same scanner and acquisition sequence, will result in different ASL image appearance and possible artefacts [20]. For example, the post-labelling delay (PLD) timing parameter controls the extent of arterial inflow into the capillary bed of the brain tissue, and so determines how well the acquired signal represents tissue perfusion rather than intravascular arterial blood volume.

Consequently, group analysis of ASL data acquired as a part of a multi-centre clinical trial is not straightforward. The main objective of this work was to determine whether ASL data acquired at a number of sites with different MRI scanners in the RADAR trial could be combined to provide consistent and clinically useful physiological information. Specifically, the feasibility of pooling data was explored with site-specific technical and protocol differences modelled as explanatory variables in the statistical analysis of the estimated grey matter CBF (CBF_GM_) data. The following hypotheses were tested:

1. Differences in ASL acquisitions result in systematic differences in CBF_GM_ which need to be accounted for when combining data from multiple sites
2. When systematic differences are accounted for, global and regional CBF_GM_ measures from multi-centre data show the expected relationships with age, sex, and scores of cognitive dysfunction
3. The spatial coefficient of variation (sCoV) of the ASL signal is a useful surrogate of brain haemodynamic status in the multi-centre context. sCoV provides a potential alternative approach for combining ASL data acquired with different acquisition parameter values

Based on the results of this work, proposals are made for the refinement of ASL data collection procedures to help direct future research and as a guide to informing protocols in future multi-centre trials.

## Methods

Detailed descriptions of the main RADAR trial and its outcomes have been published previously ([6,21]). This ASL sub-study investigated only the baseline MRI data collected prior to patient randomisation. Twenty-three NHS hospital trusts recruited AD patients for the trial; these were served by eighteen imaging centres where all participants were scanned. Of these imaging centres, nine had ASL MRI sequences available. In addition to the baseline MRI scans, the following information was collected as part of the main study: blood pressure measurements at the time of the eligibility assessment visit, baseline cognitive assessment outcomes, and demographic and clinical details.

### Participants

Participants scanned with the full neuroimaging protocol at baseline formed the cohort for this sub-study. Full trial inclusion/exclusion criteria for study participation are detailed in Kehoe et al. [21]. Briefly, participants were eligible for the trial if they were clinically diagnosed with mild-to-moderate probable Alzheimer's disease, according to the original National Institute of Neurological and Communicative Disorders and Stroke and the Alzheimer's Disease and Related Disorders Association criteria [22], and if they:

• were aged 55 years or older;
• had capacity to consent for themselves in accordance with the criteria of the UK 2005 Mental Capacity Act, as judged by trained members of the local research team;
• had a Mini-Mental State Examination (MMSE) score of 15–28;
• scored 5 or less on a modified Hachinski scale;
• had previous CT, single-photon emission computed tomography (SPECT), or MRI consistent with a diagnosis of Alzheimer's disease; and
• had a study companion who was willing to participate in the study.

Participants could participate regardless of whether they had hypertension and could already be taking licensed anti-dementia treatments and other non-renin-angiotensin system (RAS) related anti-hypertensive medications.

### Data acquisition sites and protocols

For this sub-study, all images were acquired on 3T MRI scanners. The scanning protocol consisted of:

• 3D T1-weighted whole brain structural images
• 3D T2-weighted fluid-attenuated inversion recovery (FLAIR) whole brain images, to identify white matter hyperintensities (WMHs)
• ASL images to map brain perfusion

3D T1-weighted structural images were acquired using Magnetization-Prepared Rapid Acquisition Gradient Echo (MPRAGE) on Siemens, Turbo Field Echo (TFE) on Philips, and Fast Spoiled Gradient Recalled (FSPGR) on GE Healthcare systems. Recommended imaging parameters for whole-head coverage were: repetition time (TR) = 2000ms, inversion time (TI) = 880ms, sagittal orientation, 1.0mm^3^ isotropic resolution, flip angle (FA) = 8°, parallel imaging with the acceleration factor 2.

Recommended scan parameters for the 3D T2-weighted FLAIR sequence were: TR = 5000ms, TI = 1800ms, sagittal orientation, 1.0mm^3^ isotropic resolution, Turbo/Fast Spin-Echo factor = 140, echo time (TE) = 100ms, parallel imaging with the acceleration factor 2.

Each of the scanning centres was also provided with the following recommendations regarding the ASL sequence parameters to use:

• use pseudo-continuous ASL (PCASL) if available; otherwise, PASL was also acceptable
• for PCASL, use a post-labelling delay (PLD) of ∼ 2000ms;
• for PASL QUIPSS II or Q2TIPS sequence, use a bolus duration (TI_1_) of 700ms, and an inflow time (TI_2_) of 2000ms;
• use repetition time (TR) ∼ 3500ms and minimum echo time (TE);
• acquire with transverse (axial) orientation;
• use an acquisition voxel size of 4 × 4 × 6mm^3^ and 20 slices

However, if an established ASL protocol was already in place and being regularly used at a site, the inclusion of this sequence in their RADAR MRI protocol was permitted.

### Image processing, analysis and quality control

Conversion from DICOM to NIfTI and processing of ASL was performed with ExploreASL (version 1.1.3; https://github.com/ExploreASL) [23]. Processing of 3DT1-weighted structural images included correction for white matter hyper-intensities (estimated from the FLAIR images), tissue segmentation into white matter, grey matter and cerebrospinal fluid, and spatial normalisation to MNI space [24]. The ASL processing module performed motion correction, motion outlier removal, registration with the structural images, M0 processing and CBF quantification. Quantification was done using the simplified single-compartment single-PLD model advocated in the ISMRM Perfusion Study Group consensus paper [11]. M0 calibration varied depending on the ASL MRI acquisition details: either (i) separate M0 scans, (ii) control images (when no background suppression was applied) or (iii) a single CSF M0 value was used (see Results). Mean whole brain grey matter CBF (CBF_GM_) was estimated based on the grey matter tissue segmentations. Individual image processing was carried out in each subject's native space and transformed into standard space for quality control and group analyses.

A first stage qualitative data quality assessment of the ASL images was performed via visual inspection by experienced ASL experts, and data considered to be of insufficient quality was rejected prior to analysis. Rejection criteria included a lack of labelled blood in the brain parenchyma, artefacts related to fat-shift suppression, excessive patient motion or high levels of Nyquist ghosts (see Results for examples of rejected images). To assess regional vascular effects, flow territory maps were divided into bilateral anterior, middle and posterior cerebral arteries with further division into proximal, intermediate and distal flow territories, as described previously [25]. Mean CBF and sCoV values were extracted from each region. sCoV was calculated as the ratio of standard deviation of CBF to the mean CBF in each region [33]. Partial volume correction of ASL data was not used in this study.

### Statistical analysis

All statistical analyses were performed with GraphPad Prism 9.2.0 (GraphPad Software, Boston, USA). The D'Agostino-Pearson omnibus normality test was used to check normality of the distribution of global grey matter CBF values within the sites. Differences in demographic and clinical details of patient groups scanned at different sites were assessed using one-way ANOVA with Tukey's multiple comparison test. To investigate the hypotheses of this study, the following tests were performed:

• Hypothesis 1: one-way ANOVA with Tukey's multiple comparison test and single pooled variance, to identify significant inter-site group differences in measured ASL CBF. A *p*-value of < 0.05 was considered significant.
• Hypotheses 2 and 3: to identify predictors significantly associated with measured dependent variables (CBF and sCoV) the following characteristics were entered into models of multiple linear regression: age, sex, mean arterial pressure, ADAS-Cog scores, MMSE scores, post-labelling delay during ASL MRI acquisition and ASL MRI readout module (2D vs 3D). A forced entry full model was used, as all parameters were presupposed to be relevant for physiological imaging of older adults on the verge of developing AD. The values of the sCoV of CBF were log-transformed for multiple linear regression analysis. As multiple statistical tests were concurrently conducted on several different outcomes, *p*-values were corrected for multiple comparisons with Bonferroni adjustment.

A one-way ANOVA was performed to compare independent variables (i.e., age, mean arterial pressure, ADAS-Cog scores and MMSE scores) and outcomes of interest (i.e., global and regional CBF). Tukey's multiple comparisons test, with a single pooled variance, was carried out when appropriate. A *p*-value of < 0.05 was considered significant.

## Results

### Pre-processing of brain ASL perfusion images from different sites

**Fig. 1:**
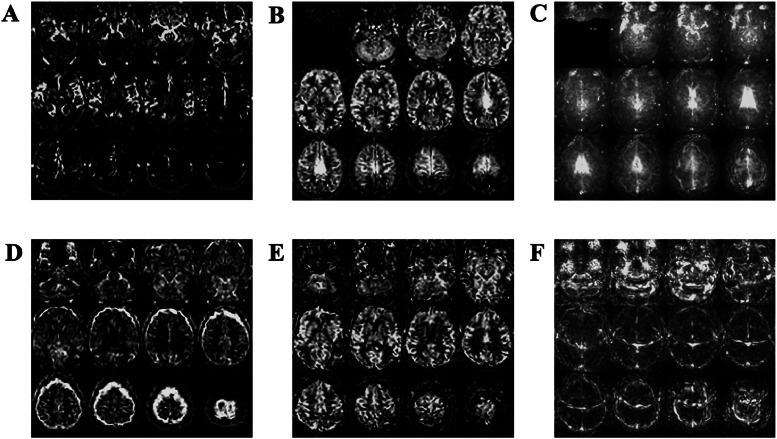
Examples of arterial spin labelling (ASL) image artefacts leading to rejection from the final analysis. (A) Tagged blood seen in the vessels but not in the tissue, commonly referred to as arterial transit artefacts (ATA) (B) Fat suppression artefact visible in the centre of the CBF image and (C) in the temporal standard deviation of CBF. (D) Motion artefact in 2D EPI images acquired without background suppression. (E) Nyquist ghost artefact present in the CBF image and (F) emphasised in the temporal standard deviation of CBF.

A total of 99 ASL data sets from 9 scanning centres were available for analysis. Of these, 27 (27%) were judged to be of insufficient quality and were rejected from the analysis. Fig. 1 shows a range of examples of rejected scans; reasons for failure to meet the qualitative threshold illustrated here were: a lack of labelled blood in parenchyma (Fig. 1A); artefacts caused by poor fat-shift suppression (Fig. 1B and C); excessive motion (Fig. 1D) or Nyquist ghosting (Fig. 1E and F); all of which rendered data sets unusable. Sample sizes were not sufficiently large to verify a normal distribution in 5 sites (*n* ≤ 5 for each), and so these data were also not taken forward for analysis.

**Fig. 2:**
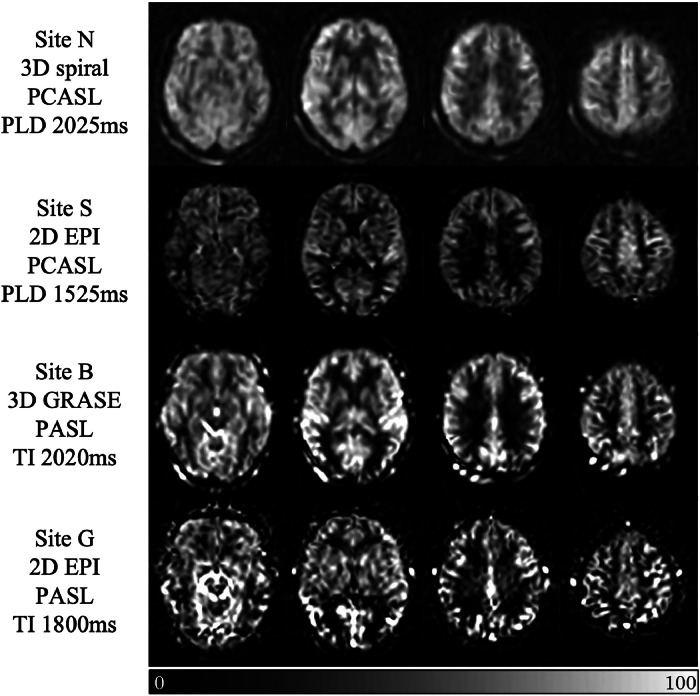
Representative CBF maps from the four sites with ASL scans used for analysis. For illustrative purposes, all images are shown in MNI space. The display window is from 0 to 100 mL/100 g/min.

Global grey matter CBF values from images acquired at the remaining 4 sites (site N (*n* = 9), site S (*n* = 12), site B (*n* = 18) and site G (*n* = 19) passed normality tests and were included in the final data set for further investigation (*n_total_* = 58). Participant information for these scanning sites is provided in Table 1, and details of the ASL acquisition parameters used are presented in Table 2. No differences were found between the sites for age, mean arterial blood pressure, ADAS-Cog scores or MMSE scores (*p* > 0.05 for all variables). Representative brain perfusion images are shown in Fig. 2, highlighting the inherently distinct characteristics of the different ASL implementations, relating to the labelling type and image readout module used.

**Table 1:** Participant information and clinical details. Data presented as Mean *±* Standard Deviation, where applicable.

	Site N (*n*=9)	Site S (*n*=12)	Site B (*n*=18)	Site G (*n*=19)
**Demographic details**				
Sex distribution	3F: 6M	5F: 7M	10F: 8M	6F: 13M
Age, years	72.3 ± 7.5	65.6 ± 8.7	68.9 ± 8.9	71.8 ± 6.0
Education, years	14.7 ± 4.4	14.4 ± 4.1	13.7 ± 3.4	12.3 ± 3.1
**AD-related data**				
Age when diagnosed	70.8 ± 7.0	64.1 ± 8.5	67.3 ± 8.8	70.6 ± 6.5
Years since diagnosed	1.7 ± 1.2	1.4 ± 0.8	1.5 ± 1.1	1.2 ± 1.4
ADAS-Cog score	18.3* ± 9.2	22.3 ± 4.3	20.8† ± 7.5	16.2 ± 4.8
MMSE score	22.9 ± 3.1	22.2 ± 4.3	21.4 ± 2.9	22.8 ± 3.0
**Medication**				
Statins, *n* (%)	4* (50.0%)	3 (25.0%)	2 (11.1%)	11 (57.9%)
Anti-platelets, *n* (%)	0 (0.0%)	2 (16.7%)	0 (0.0%)	7 (36.8%)
Psychiatric drugs∇, *n* (%)	4* (50.0%)	8 (66.7%)	4 (22.2%)	7 (36.8%)
**Blood pressure**				
Systolic, mm Hg	139.4 ±10.1	141.9 ± 17.6	137.9 ± 18.8	138.5 ± 11.2
Diastolic, mm Hg	82.9 ± 9.8	76.8 ± 10.3	76.3 ± 10.3	79.4 ± 8.3
Mean Arterial, mm Hg	101.7 ± 7.8	98.4 ± 10.8	96.9 ± 11.1	99.2 ± 7.7
**Brain volumes**				
Grey matter, ml	532.6 ± 60.7	517.6 ± 81.3	554.5 ± 70.9	557.8 ± 42.2
White matter, ml	449.5 ± 61.1	464.0 ± 92.0	464.2 ±74.2	469.0 ± 54.2
WMH, ml	50.0 ±35.3	10.7 ± 15.2	6.9 ± 10.5	14.2 ± 10.5

F = female; M = male; ADAS-Cog = Alzheimer's Disease Assessment Scale–Cognitive Sub-scale; MMSE = Mini-Mental state examination; WHM = white matter hyperintensities.

∇ Psychiatric drugs: atypical anti-psychotics (e.g., Risperidone), benzodiazepines (e.g., Lorazepam), Selective Serotonin Re-uptake Inhibitors (e.g., Citalopram), Serotonin-Norepinephrine Re-uptake Inhibitors (e.g., Duloxetine) and other antidepressants and anti-anxiety agents (e.g., Mirtazapine).

⁎ *n* = 8;

† *n* = 17

**Table 2:** ASL acquisition protocol parameters.

Site	N	S	B	G
Scanner manufacturer	GE	Philips	Siemens	Siemens
Readout module	3D stack of spirals	2D EPI	3D GRASE	2D EPI
ASL labelling scheme	PCASL	PCASL	PASL	PASL
Labelling/bolus duration (ms)	1450	1650	700	700
PLD/TI* (ms)	2025	1525	2020	1800
Echo time TE (ms)	10	12	12	11
Repetition time TR (ms)	4739	4000	3500	3500
In-plane resolution (mm)	4	3.8	3.8	4
Slice thickness (mm)	6	5.25	5.25	6
Number of slices	30	20	20	20
Parallel imaging	None	SENSE x2.5	None	GRAPPA x2
Background suppression (*n* pulses)	Yes, *n* = 5	Yes, *n* = 2	Yes, *n* = 2	Off
Scan duration (min:sec)	5:37	5:28	3:51	6:06
M0 acquisition†	Separate scan	Separate scan	Estimated from control scan	Estimated from control scan

⁎ In PASL, TI is defined as the time between the labelling pulse and the imaging excitation pulse [44]. †If estimated from the control scan, M0 is calculated using corrections for saturation recovery and/or background suppression, where appropriate.

### Hypothesis 1: Inter-site CBF comparisons

**Fig. 3:**
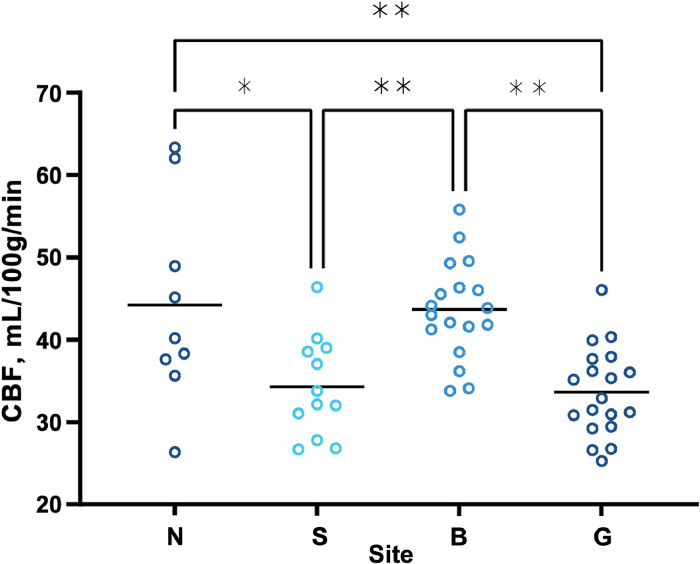
Site comparison of global grey matter brain perfusion. Circles show mean CBF_GM_ for each participant; solid black lines represent group mean values. Statistical testing by one-way ANOVA with post-hoc testing by Tukey; * *p* < 0.05, ** *p* < 0.01 (for specific p values, see text in Results).

Fig. 3 shows the estimated whole brain CBF_GM_ for all participants; there was a statistically significant difference between scanning sites (*F*(3,54) = 9.442, *p* < 0.001). A Tukey's multiple comparisons test indicated four significant comparisons. Brain perfusion values were significantly higher in data from site N (44.18 mL/100 g/min) compared to site S (34.32 mL/100 g/min, *p* = 0.014) and site G (33.63 mL/100 g/min, *p* = 0.003). Similarly, brain perfusion values were significantly higher in data from site B (43.64 mL/100 g/min) compared to site S (34.32 mL/100 g/min, *p* = 0.005) and site G (33.63 mL/100 g/min, *p* < 0.001). There was no statistically significant difference between the data from site N and site B (*p* = 0.989) or the data from site S and site G (*p* = 0.994). Interestingly, both pairs of sites with similar CBF_GM_ used scanners from different manufacturers, as well as different ASL labelling schemes. However, sites with similar CBF_GM_ used similar readout modules: sites S and G both used 2D EPI, whereas sites N and B both used 3D readouts.

### Hypothesis 2: Combining inter-site CBF data to identify perfusion modifiers

**Fig. 4:**
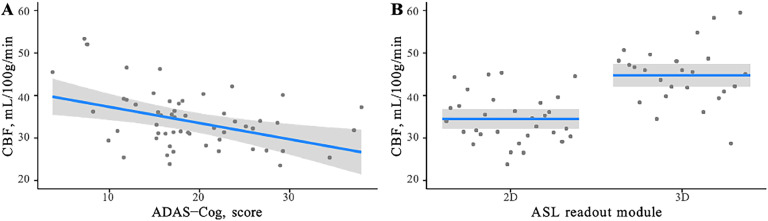
Multiple linear regression results for significant brain perfusion predictors: (A) ADAS-Cog score and (B) ASL readout module type.

A multiple regression analysis was conducted to determine if demographic and clinical variables were associated with brain perfusion when differences in PLD/TI and readout module (2D vs 3D) were accounted for. The data was assessed for collinearity of regressors, and no evidence of multi-collinearity was found (all variance inflation factor (VIF) values < 5). Furthermore, the data satisfied the assumption of independent errors, as indicated by a Durbin-Watson value of 2.06. The results indicated that 47.6% of the variance in the data could be explained by the predictor variables (*R*^2^ = 0.40, *F* (7, 48) = 6.23, *p* < 0.001, Table 3). ADAS-Cog score emerged as a significant modifier and explained 8.5% of the grey matter CBF variability (Fig. 4A). More significantly, 35.6% of the variance was explained by the ASL readout module, where CBF values obtained using a 3D readout (spiral or GRASE) were found to be significantly higher than those obtained using 2D EPI readout (Fig. 4B).

**Table 3:** Factors affecting global CBF_GM_ identified using multiple linear regression. Values shown indicate regression coefficients and their 95% confidence intervals (CI).

Independent variable	Estimate	95% CI		Goodness of fit
		*Lower*	*Upper*	
Intercept	30.27	−1.68	62.21	
Age, years	−0.03	−0.25	0.20	
Sex (reference: male)	−2.56	−6.35	1.22	*R^2^* = 0.48
MAP, mm Hg	0.08	−0.13	0.28	*R^2^_adj_* = 0.40
ADAS-Cog, score	−0.38*	−0.72	−0.04	*R^2^_pred_* = 0.25
MMSE, score	0.13	−0.62	0.89	
PLD, seconds	3.03	−3.70	9.77	
ASL readout module (ref: 3D)	**9.36**†	5.22	13.50	

⁎ *p* < 0.05,.

† *p* < 0.001.

**Fig. 5:**
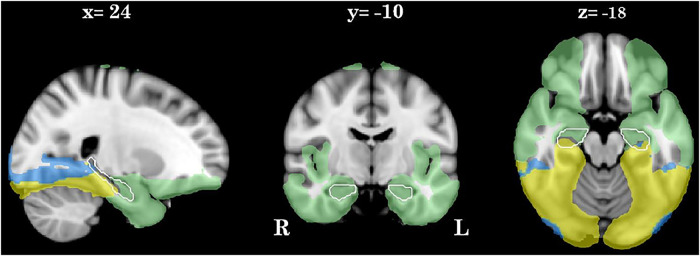
Flow territories where perfusion was linked with ADAS-Cog score. Flow territories perfused by bilateral proximal middle cerebral arteries (green), proximal posterior cerebral arteries (yellow) and intermediate posterior cerebral arteries (blue). Coordinates for sagittal, coronal and axial slices are in MNI152 space. Outlines of the anatomical masks of hippocampi, defined using the Harvard-Oxford cortical structural atlas, are overlaid in white. After applying Bonferroni adjustment for multiple comparisons, only the associations between blood flow values in territories perfused by the bilateral posterior cerebral arteries (proximal and intermediate; yellow and blue) remained statistically significant in relation to ADAS-Cog scores.

To further investigate the region-specific effect of perfusion modifiers, the analysis was repeated with the CBF_GM_ values from flow territories based on feeding arteries (Table 4). The data was assessed for collinearity of regressors, and the results indicated no evidence of multi-collinearity (all VIF values < 5). All multiple linear regression models satisfied the assumption of independent errors, as indicated by Durbin-Watson values ranging from 1.66 to 2.17. Perfusion values in distal regions were most dependent on the PLD used, and lower CBF_GM_ values in territories perfused by the bilateral proximal and intermediate posterior cerebral arteries were associated with higher ADAS-Cog scores. The regional results regarding ADAS-Cog scores are illustrated in Fig. 5.

**Table 4:** Factors affecting CBF_GM_ in different flow territories, identified using multiple linear regression. Values shown indicate regression coefficients and their 95% confidence intervals (CI). ACA = bilateral anterior cerebral arteries; MCA = bilateral middle cerebral arteries; PCA = bilateral posterior cerebral arteries. Reference for gender - male; reference for ASL readout - 3D.

Independent variable	ACA							MCA							PCA				
	Estimate		95% CI					Estimate		95% CI					Estimate		95% CI		
			*Lower*		*Upper*					*Lower*		*Upper*					*Lower*		*Upper*
***Proximal flow territories***																			
Intercept	30.90		−7.58		69.38			29.58		1.10		58.05			58.17*		20.17		96.17
Age, years	0.06		−0.20		0.33			0.14		−0.06		0.34			0.13		−0.13		0.40
Sex (ref: male)	−0.63		−5.18		3.93			−0.58		−3.95		2.80			−1.30		−5.80		3.20
MAP, mm Hg	−0.01		−0.26		0.23			0.001		−0.18		0.18			0.06		−0.18		0.31
ADAS-Cog, score	−0.20		−0.61		0.21			−0.40		−0.70		−0.09			**−0.78**§		−1.18		−0.37
MMSE, score	0.33		−0.58		1.23			−0.07		−0.74		0.60			−0.43		−1.33		0.47
PLD, seconds	0.51		−7.60		8.63			3.08		−2.92		9.08			−7.73		−15.74		0.29
ASL readout module (ref: 3D)	**11.27**†		6.29		16.25			6.02*		2.33		9.71			6.57		1.65		11.49
Goodness of fit	*R^2^* = 0.39							*R^2^* = 0.39							*R^2^* = 0.39				
	*R^2^_adj_* = 0.31							*R^2^_adj_* = 0.30							*R^2^_adj_* = 0.30				
	*R^2^_pred_* = 0.16							*R^2^_pred_* = 0.14							*R^2^_pred_* = 0.17				
***Intermediate flow territories***																			
Intercept	10.83		−28.82			50.48		4.05	−38.40		46.51			49.28		12.53		86.04	
Age, years	−0.003		−0.28			0.27		0.07	−0.22		0.37			−0.08		−0.34		0.17	
Sex (ref: male)	−4.05		−8.75			0.64		−3.01	−8.04		2.02			−3.88		−8.23		0.48	
MAP, mm Hg	0.09		−0.17			0.34		0.11	−0.16		0.38			0.13		−0.11		0.36	
ADAS-Cog, score	−0.32		−0.75			0.10		−0.20	−0.66		0.25			−0.64*		−1.03		−0.24	
MMSE, score	0.69		−0.24			1.63		0.85	−0.16		1.85			−0.12		−0.99		0.74	
PLD, seconds	7.75		−0.61			16.11		1.87	−7.09		10.82			−3.61		−11.37		4.14	
ASL readout module (ref: 3D)	8.58*		3.45			13.72		**11.67**§	6.17		17.17			**12.16†**		7.40		16.92	
Goodness of fit	*R^2^* = 0.45							*R^2^* = 0.42						*R^2^* = 0.51					
	*R^2^_adj_* = 0.37							*R^2^_adj_* = 0.34						*R^2^_adj_* = 0.44					
	*R^2^_pred_* = 0.22							*R^2^_pred_* = 0.15						*R^2^_pred_* = 0.32					
***Distal flow territories***																			
Intercept	−1.39	−43.17		40.39			−9.81			−48.48			28.87	8.21		−35.63		86.04	
Age, years	−0.05	−0.34		0.25			0.06			−0.21			0.33	−0.20		−0.51		0.10	
Sex (ref: male)	−3.80	−8.74		1.15			−4.35			−8.93			0.23	−6.28		−11.47		−1.09	
MAP, mm Hg	0.12	−0.15		0.39			0.13			−0.12			0.38	0.16		−0.12		0.44	
ADAS-Cog, score	−0.30	−0.75		0.15			−0.38			−0.79			0.04	−0.38		−0.85		0.09	
MMSE, score	0.65	−0.33		1.64			0.69			−0.23			1.60	0.65		−0.39		1.68	
PLD, seconds	13.84*	5.03		22.65			14.69*			6.53			22.84	10.19		0.95		19.44	
ASL readout module (ref: 3D)	−3.18	−2.23		8.59			2.19			−7.20			2.82	6.02		0.34		11.69	
Goodness of fit	*R^2^* = 0.38						*R^2^* = 0.39							*R^2^* = 0.42					
	*R^2^_adj_* = 0.29						*R^2^_adj_* = 0.30							*R^2^_adj_* = 0.33					
	*R^2^_pred_* = 0.09						*R^2^_pred_* = 0.11							*R^2^_pred_* = 0.20					

⁎ *p* < 0.05.

§ *p* < 0.01,.

† *p* < 0.001, with Bonferroni adjustment for multiple comparisons.

### Hypothesis 3: Spatial CoV of CBF as a surrogate haemodynamic parameter

**Fig. 6:**
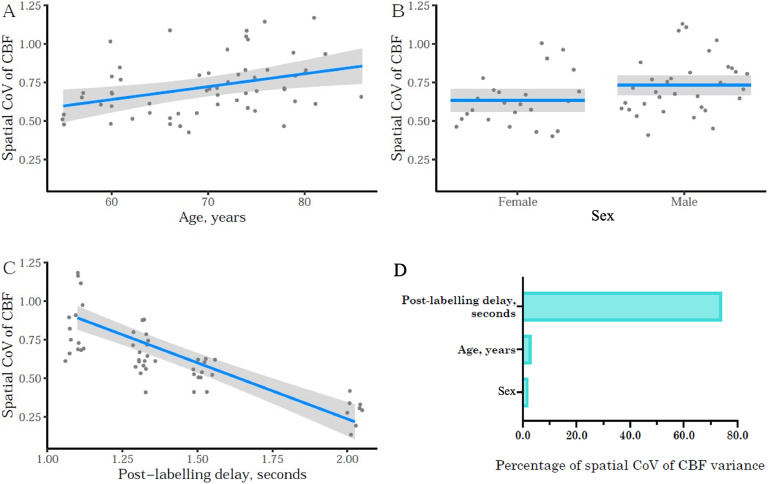
Multiple linear regression results for sCoV of CBF, demonstrating associations with (A) age, (B) sex and (C) PLD/TI of the ASL sequence. (D) Visual representation of the relative contributions of each predictor to the *R*^2^.

A separate multiple regression analysis was conducted to investigate the relationship between the sCoV of CBF and clinical variables of interest. Multiple-collinearity was not observed (VIF values ranged from 1.02 to 1.90), and the assumption of independent errors was satisfied (Durbin-Watson value of 2.19). To account for the simultaneous multiple linear regression analysis conducted on two different outcomes (i.e. CBF and sCoV of CBF), Bonferroni correction was used to adjust for multiple comparisons. The coefficient of determination *R*^2^ was 0.82, while predictive *R*^2^ was 0.75, indicating that 75% of the performance of the model was explained by the included factors, with 7% of the model attributable to random correlations or other factors (Table 5 and Fig. 6). 74.2% of variance in sCoV of grey matter CBF could be explained by the PLD values, while age and sex together explained 5% of variation (Fig. 6D).

**Table 5:** Mulitple linear regression coefficients for the sCoV of global gray matter CBF. A strong linear association of sCoV with PLD was observed (see also Fig. 6), along with weaker but significant associations with age and sex. The analysis was performed with the log-transformed values of the sCOV; the reference for sex was male and the reference for ASL readout module was 3D.

Independent variable	Estimate	95% CI		Goodness of fit
		*Lower*	*Upper*	
Intercept	**2.574**†	2.089	3.059	
Age, years	0.005*	0.001	0.008	*R^2^* = 0.82 *R^2^_adj_* = 0.79 *R^2^_pred_* = 0.75
Sex	0.074*	0.016	0.131	
MAP, mm Hg	−0.002	−0.005	0.001	
ADAS-Cog, score	0.001	−0.004	0.006	
MMSE, score	−0.007	−0.019	0.004	
PLD, seconds	**−0.597**†	−0.699	−0.494	
ASL readout module	−0.023	−0.086	0.040	

⁎ *p* < 0.05,.

† *p* < 0.001.

## Discussion

In a sample of subjects with mild-to-moderate Alzheimer's disease, recruited as part of a blinded phase II randomised controlled trial, global CBF_GM_ measured using PCASL and PASL across 4 different sites ranged between 25 mL/100 g/min and 63 mL/100 g/min. This range of values is comparable with other studies investigating AD cohorts [[26], [27], [28], [29]]. The participant groups did not differ in age between sites, and there were no significant differences in their cognitive status, as measured by ADAS-Cog and MMSE, consistent with the overall findings of the RADAR trial in which the primary outcome was also not realised [21]. However, CBF_GM_ measurements varied significantly between the research sites.

The pooled data analysis revealed that ASL readout (2D vs 3D) accounted for 35.6% of variance in brain perfusion estimates, suggesting that the differences between sites primarily reflected data acquisition differences in the ASL MRI protocols rather than disease-related physiological differences between the participants. At a group level, CBF_GM_ measurements were linked to the ADAS-Cog score, in concordance with previous studies [[30], [31], [32]]. This suggests that after regressing out the effects of important acquisition protocol differences (i.e. ASL readout module, PLD), ASL can successfully be used in a multi-centre setting to address biological research questions of this nature.

Furthermore, the analysis examining the sCoV of CBF revealed significant associations with age and sex, consistent with findings from a previous study [33]. However, it is important to note that the sCoV values were not found to be associated with the level of cognitive impairment in this study. The relationship between spatial heterogeneity of CBF_GM_ and cognitive decline or dementia has been investigated in several studies, with inconsistent results. Some studies have reported positive associations [[34], [35], [36]], while others have found less conclusive results [37].

The observed CBF_GM_ differences across different sites in this study (e.g. as illustrated in Fig. 2) were at least in part due to the range of PLD values used, as shorter PLD resulted in more prominent vascular artefacts. To assess the interaction of variable bolus arrival times and PLD, an exploration of distinct flow territories was carried out, based on the known vascular anatomy. As expected, perfusion in distal regions of the brain was affected most by variation in PLD. Furthermore, it was observed that lower CBF_GM_ values in territories perfused by the bilateral proximal and intermediate posterior cerebral arteries were linked with higher ADAS-Cog scores (i.e. greater cognitive dysfunction). As the hippocampal arteries arise predominantly from the posterior cerebral artery [38], such a relationship is in concordance with the literature reporting lower perfusion values in the hippocampal region in AD patients compared with healthy controls ([28,39]). Although the evidence to support the effect of sex on CBF_GM_ was not substantial in this study, likely due to the restricted sample size, a subtle effect of lower brain perfusion was seen in men compared to women in both global and ROI measures. Perfusion analysis results did not demonstrate significant relationships with age or mean arterial blood pressure within this cohort.

In this study, a notable substantial portion of the variability (74.2%) in ASL sCoV was attributed to the differing PLD values, despite the quantification procedure theoretically accounting for this. These findings align with a previous study [40] which also demonstrated the influence of scanning parameters, particularly PLD, on PCASL sCoV. Despite the influence of variations in PLD, the associations observed between sCoV and the main CBF modifiers, such as age and sex, suggest that sCoV can serve as a useful proxy for haemodynamic status. These associations indicate the potential for sCoV to be utilised in combination with CBF as a meaningful physiological measure in multi-centre ASL studies.

### Study limitations

A real-world clinical research study inevitably needs to make compromises regarding study design and data compatibility. In RADAR, a variety of scanners from different manufacturers were used (Philips, Siemens, GE), with inherently different hardware and software systems. Across all the participating RADAR sites, it was not always possible to implement the recommended imaging protocols. Moreover, while ASL data were acquired at nine research sites, data from only four of these sites were included in the analysis, due to low numbers of participants and quality control failures at some of the other sites. Consequently, the limited amount of data restricted the ability to account for technical differences across the different sites and to identify potential sources of variability in the data sample.

Furthermore, the ASL inflow times utilised at several sites deviated slightly from the recommended value outlined in the ASL ‘white paper’ (i.e. 2000ms) [11]. In this study, PCASL PLD values of 1525ms and 2025ms, and PASL TI_2_ values of 1800ms and 2020ms were employed. Short PLD can result in incomplete delivery of the ASL labelled bolus to the brain, leading to quantification errors and strong vascular artefacts [11]. Including PLD as a regressor in the multi-centre analysis enabled estimation of the effect size of this variability, and allowed it to be factored out. While this study incorporated both PLD and the ASL readout module into the multi-centre ASL CBF regression model, future studies should investigate additional parameters relating to scanning protocols. These parameters may include the background suppression method, the M0 estimation approach, the parallel imaging acceleration factor, and the relative positioning of the ASL labelling and imaging regions.

Age- and disease-related alterations in tissue volumes also have the potential to affect the accuracy of CBF quantification. It is important to note that estimating global grey matter volume relies on automatic segmentation and registration algorithms. However, these algorithms may not be optimally efficient in individuals with Alzheimer's disease, where regional brain atrophy occurs. Partial volume correction was not applied as part of the quantification process, as the benefit of doing this is currently uncertain [41]. CBF quantification is also dependent on the accuracy of M0 estimation, and in this study one of three different methods was employed, depending on the site: a separate M0 image was acquired in sites N and S; a single numerical value was used for site B; and in site G, the control images from ASL MRI were utilised. This depended on the ASL pulse sequence implementation and the available options on each scanner.

While no significant differences of the main demographic and clinical characteristics were found among participants from different sites, it is still possible that these factors influenced the results of this study. To try to more specifically isolate inter-site differences and biases in the ASL measurements caused by methodological disparities, a ‘travelling volunteer’ study could be performed, in which a group of the same individuals is scanned at all sites. While this would address cohort variability to some extent, the practical difficulties of arranging and coordinating scanning sessions for a cohort of volunteers with the appropriate demographic characteristics are challenging. In addition, due to the geographical spread of the sites, scanning would need to occur on different days, potentially some time apart, which can introduce variability relating to difficult-to-control physiological factors [42]. Consequently, we did not explore this approach, and kept our analysis to the participants in the RADAR trial.

### Recommendations for future studies

The data presented here highlight several observations of issues that might hinder the successful utilisation of ASL MRI in a multi-centre study. To improve future investigations of brain perfusion, we offer the following recommendations for procedures of data acquisition and quality assessment, and specifications in the MRI protocol.

First, a comprehensive review of the ASL sequences and readouts available in the participating MRI centres prior to the start of the trial is highly beneficial, so that the highest degree of matching can be achieved. Indeed, in the event there might be a larger pool of sites than may be needed to deliver the recruitment target then selection of sites based on strong matching potential would be a worthwhile enhancement. Second, strong adherence by participating sites to a well-defined and cohort-appropriate MRI scanning protocol is highly desirable. In situations where scanning parameters cannot be set to the values advised (due to the operating system software or pulse sequence differences), parameter selection should be agreed centrally and not at a single site level. A rigorous quality assessment of any available demographically matched healthy volunteer pilot data and the initial study participants at each site is highly recommended. Doing this using a pilot stage, or prior to a site being fully opened to recruitment, provides the possibility of making scanning protocol adjustments relevant to the population of interest before the main study is fully underway. For consistency, this data quality assessment should be carried out by the designated personnel overseeing all scanning sites.

Another potential stand-alone hypothesis-driven question, which could be served by either a single site study with a large clinical cohort, or a multi-centre study with sufficient volumetric data, was the curious observation of apparently higher brain volumes (i.e. indicating less deterioration) in sites where there was higher statin use. A possible explanation for this is that statin use may have had a protective effect in stabilizing amyloid-related pathology, which could translate to lower rates of atrophy [43]. However, considering the number of analytical variables involved, including different statin dosages (0, 10, 20 and 40mg) and small sample sizes (*n* = 11 and *n* = 4) for the number of individuals taking statins, the scientific value of a sub-analysis in this study would be limited. However, a larger, more statistically powered cohort could properly interrogate and provide further replication and validation of the observations previously reported by Nabizadeh and colleagues in the Alzheimer's Disease Neuroimaging Initiative (ADNI) [43].

The most suitable implementation of ASL MRI for clinical applications is covered in detail in the ASL White Paper [11]. In addition to the labelling parameters outlined there, other details should be defined in the documentation provided to the MR scanning centres. For example, this information should include details such as the number of signal averages required, labelling plane positioning (where appropriate) and how the M0 images should be acquired.

## Conclusion

Inherent protocol variability in multi-centre ASL scanning leads to significant differences in the resulting perfusion parameter maps. While protocol harmonisation is recommended, it is important to recognise that this can only be achieved to a limited degree, especially when multiple scanner types are included in a study. Multiple linear regression can be used to account for variability in acquisition techniques and parameters, and by employing this approach we were able to identify an association between CBF_GM_ and ADAS-Cog scores in the RADAR cohort of patients with mild-moderate AD, particularly in regions supplied by the posterior cerebral arteries. In addition, the sCoV of CBF exhibited links with age and sex. While clearly revealing the challenges of using ASL for multi-centre dementia studies, this work also highlights the valuable haemodynamic information that can be made available through this scanning modality, aiding the answering of important mechanistic questions but also emphasising the need for careful protocol design, set-up and monitoring over the duration of the study.

## CRediT authorship contribution statement

**Lina Jarutyte:** Writing – review & editing, Writing – original draft, Visualization, Investigation, Data curation, Conceptualization. **Jan Petr:** Writing – review & editing, Formal analysis, Data curation, Conceptualization. **Nicholas Turner:** Writing – review & editing, Project administration, Formal analysis, Data curation. **Patrick G. Kehoe:** Writing – review & editing, Supervision, Project administration, Methodology, Investigation, Funding acquisition, Conceptualization. **Henk-Jan Mutsaerts:** Writing – review & editing, Supervision, Formal analysis, Data curation, Conceptualization. **David L. Thomas:** Writing – review & editing, Writing – original draft, Supervision, Project administration, Investigation, Formal analysis, Data curation, Conceptualization.

## Declaration of competing interest

The authors declare the following financial interests/personal relationships which may be considered as potential competing interests:

Patrick G Kehoe reports financial support was provided by Efficacy and Mechanism Evaluation Programme (NIHR). Henk-Jan Mutsaerts reports financial support was provided by Horizon Europe. Henk-Jan Mutsaerts and Jan Petr report financial support was provided by eScience Open eScience Call (OEC). Henk-Jan Mutsaerts and Jan Petr report financial support was provided by Dutch Heart Foundation. Henk-Jan Mutsaerts and Jan Petr report financial support was provided by Joint Program Neurodegenerative Disease (JPND). Henk-Jan Mutsaerts reports financial support was provided by Eurostars. If there are other authors, they declare that they have no known competing financial interests or personal relationships that could have appeared to influence the work reported in this paper.
